# A New Method for Inferring Hidden Markov Models from Noisy Time Sequences

**DOI:** 10.1371/journal.pone.0029703

**Published:** 2012-01-11

**Authors:** David Kelly, Mark Dillingham, Andrew Hudson, Karoline Wiesner

**Affiliations:** 1 School of Mathematics, University of Bristol, Bristol, United Kingdom; 2 School of Biochemistry, University of Bristol, Bristol, United Kingdom; 3 Department of Chemistry, University of Leicester, Leicester, United Kingdom; 4 School of Mathematics, University of Bristol, Bristol, United Kingdom; Universita' del Piemonte Orientale, Italy

## Abstract

We present a new method for inferring hidden Markov models from noisy time sequences without the necessity of assuming a model architecture, thus allowing for the detection of degenerate states. This is based on the statistical prediction techniques developed by Crutchfield et al. and generates so called causal state models, equivalent in structure to hidden Markov models. The new method is applicable to any continuous data which clusters around discrete values and exhibits multiple transitions between these values such as tethered particle motion data or Fluorescence Resonance Energy Transfer (FRET) spectra. The algorithms developed have been shown to perform well on simulated data, demonstrating the ability to recover the model used to generate the data under high noise, sparse data conditions and the ability to infer the existence of degenerate states. They have also been applied to new experimental FRET data of Holliday Junction dynamics, extracting the expected two state model and providing values for the transition rates in good agreement with previous results and with results obtained using existing maximum likelihood based methods. The method differs markedly from previous Markov-model reconstructions in being able to uncover truly hidden states.

## Introduction

Recent advances in experimental techniques have given new insight into many molecular systems, often on the single molecule level [1]–[5]. However, the data yielded from experiments at this cutting edge are frequently beset by noise which makes quantitative analysis difficult. The analysis of Fluorescence Resonance Energy Transfer (FRET) spectra is a typical example of this problem.

FRET spectroscopy is a powerful method for investigating systems such as DNA molecules since it is unique in its sensitivity to molecular conformation, association, and separation in the 1–10 nm range. It allows the dynamics of single molecules to be observed, avoiding the averaging inherent in ensemble measurements. In FRET spectroscopy, energy is transferred non-radiatively via a long range dipole-dipole interaction from one fluorophore to another, strategically attached to different parts of the molecule(s) under study. The efficiency of this energy transfer is strongly modulated by the separation, 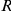, of the fluorophores, with a 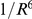 dependence and so is highly sensitive to changes in conformation or association. For a more detailed description of the principles and techniques of FRET spectroscopy see, for example, Jares-Erijman et al. [6] and Ha et al. [7] and references therein.

Since transitions between different conformational states typically take a time shorter than the resolution of the measurement, one might expect FRET spectra to exhibit jumps between discrete values (FRET efficiency levels). However, there are many sources of instrumental noise and also photophysical effects and temporal coarse graining. These result in the distribution of the data around some mean value, obscuring the underlying dynamics, especially in systems with many FRET levels. The sources of noise have been discussed by a number of groups [8]–[10]. As the systems investigated *via* FRET spectroscopy have become more complicated, a need for objective data analysis methods has been recognised. Hidden Markov Models (HMMs) are a good choice for modeling the conformational dynamics of systems. Methods of inference are well understood and the states can be interpreted as conformational states of molecules or particular associations between molecules.

However, establishing the correct model architecture (the number of states in the model and the transitions between them) is a challenge. In choosing a model architecture, we must compromise between maximising the likelihood of the observations given the model and minimising the model size. It can be done using the Bayesian or Akaike Information Criteria. This is the approach taken by McKinney et al [11] in prior work addressing this very problem. In their work, efficient algorithms were developed for finding model parameters which maximised the model likelihood. Then the number of states in the model was adjusted based on the average occupancy of each state, with states which were rarely visited being removed to simplify the model with only small reductions in model likelihood. These algorithms, however, can only infer Markov chains of varying order and are not able to detect hidden states.

We present here an alternative method, based on statistical prediction techniques, which can detect hidden states. It uses the same principles of maximising model likelihood and parsimony and is applicable not only to FRET spectra but to any noisy time sequence displaying the following properties. Firstly the data must be clustered around discrete values. Secondly these discrete values must be sufficiently separated relative to the variance and quantity of the data (this will be explained in more detail below). Thirdly, there must be sufficient examples of switching (transitions) between these discrete values. Finally, the statistics of these transitions must be stationary, that is, the transition probabilities and the distribution of the observations must be constant with time. (We note that existing methods of analysis implicitly make the same assumption of stationarity. This assumption is discussed in the supporting information (S.I.), Text S1, Section 4 and Figs. S5, S6, S7, along with suggested methods to check its validity).

This method has the advantage that it is capable of inferring the existence of degenerate states, states associated with the same discrete value. In the context of FRET spectra, it is not necessary to associate one state with one FRET efficiency level (as is done by McKinney et al.), degenerate levels may also be discovered if revealed by the structure of the transitions between levels. In addition, the methods offer comparable performance in terms of speed and ease of use to existing model inference methods and remove the potential source of subjectivity of the selection of model architecture.

First, we will outline the theory of causal state models and the challenges to be overcome in applying such techniques to noisy time sequences. Then we shall describe the new method and the results of its application to simulated FRET spectra. Finally, we will illustrate the use of the method on the study of Holliday Junction conformational dynamics and compare this with the method of McKinney et al.

### Causal State Models

Causal state models [12] are equivalent to HMMs in their structure; they both consist of a number of states connected by transitions described by a transition probability matrix and have some output (such as a real number sampled from a distribution) associated with each transition.

However, causal state models differ from HMMs in that the states represent the structure or regularities present in the data. These states are so-called *causal states*; equivalence classes which group together past subsequences which share the same conditional distribution of future subsequences. In this way, if one knows what causal state a process is in, one can make as informed an estimate of the future of the process as is possible. The set of causal states is a sufficient statistic, encapsulating the same amount of information relevant to the future of the process as the entire past data sequence.

To put this in more mathematical terms, let us define a bi-infinite sequence of discrete random variables representing a stationary data sequence, 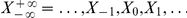, and a particular realisation as 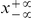. Then the past and future at time 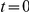 are denoted 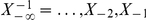 and 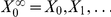 respectively and their realisations 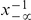 and 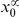.

The condition of the equivalence relation, 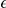, is then expressed as
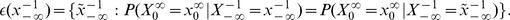
(1)


Note that the stationarity assumption is an important one, since the future distributions of past subsequences must be constant if we are to be able to use them for prediction.

Let 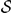 be the set of causal states generated from these equivalence classes. The Excess Entropy, 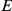, is defined as the mutual information between the past and future of the sequence, where mutual information has its usual definition, see, for example, Cover and Thomas [13]. Due to the sufficiency of the causal states the following is true [14]

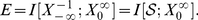
(2)


In the case of infinite data, a model based on causal states is provably a unique, minimal, optimal, statistical predictor of the future of the data sequence [12], [14]–[16]. The proofs of the uniqueness, minimality and optimality of this statistic are outside of the scope of the current work but the interested reader is referred to the original papers.

In reality, data is finite and so we must estimate the causal states based on available data. This necessitates two compromises. Firstly, the length of the past subsequences comprising the causal states must be limited such that the frequency with which the longest past subsequences are observed is sufficient to estimate the distribution of future subsequences with reasonable confidence. Secondly, the distributions of future subsequences conditioned on different pasts (e.g. 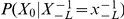 and 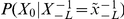 where 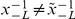) which would be equal in the limit of infinite data (if drawn from the same underlying distribution) will be so no longer and so a statistical test is required to determine equivalence at some chosen significance level. These practical constraints mean that there are two parameters which must be chosen, the maximum length of subsequence examined, 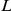, and the test significance level, 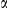. However the size of the data set, 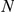, and the significance level together allow the maximum reasonable length of subsequence to be determined given the sensitivity of the statistical test.

Once the estimated causal states have been determined they may be linked to form an HMM by appending each of the past subsequences in the causal states with each symbol from the alphabet. The transition is determined by finding the causal state containing the resulting subsequence, with the transition probabilities determined by the relative frequencies of the new subsequences. Since the HMM must be deterministic (the observation of a symbol when occupying a certain state must uniquely determine which state is transited to) the causal states may be split until a deterministic HMM is found. This procedure has been implemented as the Causal State Splitting Reconstruction (CSSR) algorithm by Shalizi and Shalizi [17].

Causal State models have been successfully applied to many systems including spin systems [18], crystal growth [19], molecular dynamics [20], atmospheric turbulence [21], population dynamics [22], [23], and neural spike sequences [24].

### Application to FRET Spectra

Data in the real world is rarely discrete. The discrete data upon which these causal state methods are based is assumed to have been observed via some measurement channel with a finite resolution. Obviously, the HMM obtained is strongly dependent on this resolution. If we are to apply these methods to FRET spectroscopy, we wish our resulting HMM to be independent of the discretisation scheme used to obtain it, since for the model to be useful it should be determined by the underlying system, not by the particulars of the method used to obtain it.

FRET spectra would ideally be discrete since the system undergoes transitions between conformational states corresponding to certain FRET efficiencies on a timescale shorter than that of observations, resulting in discrete jumps between FRET levels. It is a natural choice, therefore, to base any discretisation scheme on these FRET levels.

However, there are many experimental sources of noise which result in data being 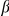-distributed (or to a reasonable approximation normally distributed) around the idealised FRET levels with distributions typically overlapping [25]. This noise in spectra makes it impossible to determine with certainty to which FRET level each data point should belong. Misassignment of FRET levels distorts distributions and introduces fallacious structure which, in the case of simulated data, leads to inferred HMMs varying from the models used to generate the data.

The methods presented in the next section address this problem, allowing the identification of a minimal representation of the dynamical structure hidden within the data.

## Methods

In contrast to conventional methods (which typically ignore uncertainty in assignments), explicitly recognising uncertainty in the discretisation allows the problem of noise to be circumvented. By assigning a special null symbol to any data point which could not be reliably assigned to a FRET level and then disregarding these symbols when determining causal states, the underlying model architecture (that used to generate the data in the case of simulated spectra) can be inferred.

**Figure 1:**
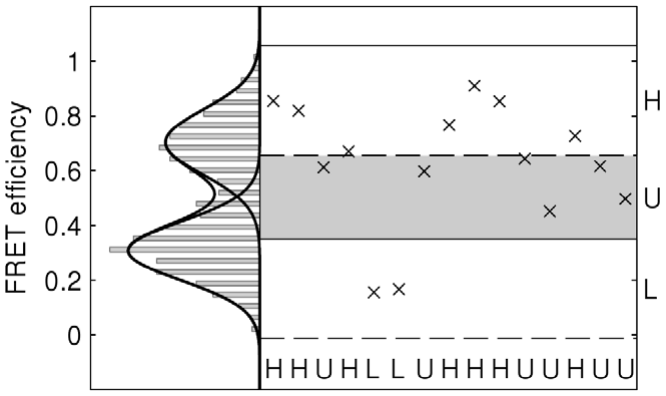
Illustration of partition scheme. On the vertical axis the histogram of the spectrum is shown, along with the fitted Gaussian mixture model. The resulting partitions are shown with solid horizontal lines where the upper component's probability reaches 0.001 and dashed lines for the lower component. A short section of the spectrum is also shown with the corresponding symbol sequence. Here H and L correspond to the high and low FRET levels respectively and U indicates uncertainty.

The procedure (illustrated in Fig. 1) is as follows;

1. Construct a histogram of FRET efficiencies.
2. Fit Gaussian mixture models with varying numbers of components. (Note that Gaussian mixture models are used since FRET levels are believed to be well approximated by Gaussian distributions, as mentioned above.)
3. Select a mixture model using the Akaike Information Criterion. (As pointed out by a referee, the Akaike information criterion has been known to overfit in certain circumstances [26]. We found it performed satisfactorily for this application but users should be aware of the issue. The Bayesian information criterion could equally well be used.)
4. Partition the space. For a model with 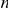 components there will be 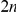 partition boundaries, located where the probability of observing a data point generated by each model component reaches some small, user defined limit (i.e. the permille quantiles). There will be 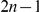 bounded regions defined by these boundaries.

The partition boundaries associated with each model component may or may not overlap with partitions associated with other model components depending on the separation of the means relative to the variances. In either case the odd numbered regions correspond to certain assignment of data points to one model component. The even numbered regions in between correspond to regions of uncertainty. Here there is a non-negligible probability of a data point being generated by more than one model component, either because model components overlap or because the probability of a data point being generated by any component is very low.

Note that this partitioning assumes that the partitions associated with any one model component do not both fall in between the partitions associated with another, an unlikely circumstance which could only occur with FRET levels extremely close together or with very different variances. If this does occur, appropriate partitions cannot be found.

1. For each model component, part of it lies within one partition (associated with certain assignment of data to that model component) and the remaining portion lies within another partition (associated with uncertainty). Calculate the fraction of the probability mass associated with certain assignment for each model component. Find the minimum of these and adjust the other partition boundaries in order to equalise them. For an example of this see the S.I., Text S1, Section 1 (Figs. S1, S2, S3, S4).

The reason for this is that this partitioning effectively discards a proportion of the occurrences of each possible subsequence in the discretised data. If we discard more of one subsequence than another we skew their relative frequencies and, as a result, alter the transition probabilities of the HMM. By maintaining the original ratios between model components in the partitioning we avoid this source of bias. A proof of this is included in the S.I., Text S1, Section 2.

1. Assign each data point a symbol based on the partition in which it lies. Points which were generated by one component of the mixture model with high probability (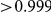) are assigned the symbol corresponding to this component. Points located where there is any overlap of components are assigned the null symbol.
2. Determine the causal states of the model using an adapted version of the CSSR algorithm. The adaptation is to only append symbols which are certain to existing subsequences (starting with the empty subsequence) so subsequences containing the null symbol are never considered. The CSSR algorithm is described in detail by way of an example in the S.I., Text S1, Section 3 (Tables S1, S2, S3, S4).

Since the distribution of FRET efficiencies is such that there is a non-zero probability of observing a data point far from the mean, there is still a small probability of misassignment of data points. If this occurs there may be extra transitions present in the inferred HMM, however the probability of these transitions is generally very small relative to other transitions present and as such may be easily identified. There is necessarily a compromise between obtaining a sufficient proportion of non-null symbols to be able to determine the causal states and avoiding misassignment. The location of the partitions with regards to this compromise will be dictated by the data; it is easier to avoid misassignments where the FRET levels are widely spaced. These methods have been implemented in Matlab (available online at http://www.mathworks.com/matlabcentral/fileexchange/33217).

## Results

### Simulated Data

**Figure 2:**
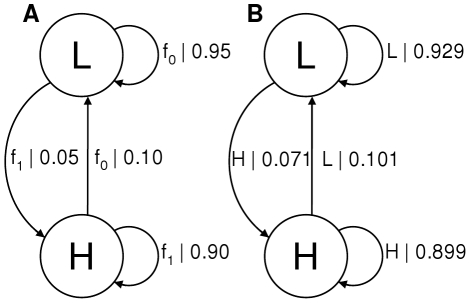
Comparison of generating and inferred HMMs. A) The HMM used to generate the data and B) the HMM inferred from the data. For the generating model the transitions are labelled with the function sampled to generate a data point and its probability. For the inferred model the transitions are labelled with the symbol output on the transition and its probability.

We demonstrate the algorithm with simulated FRET data. A typical FRET system was simulated using the HMM shown in Fig. 2. Rather than outputting a particular symbol on each transition, a Gaussian function, 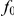 or 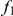, was sampled. The means of the two functions were 0.3 and 0.7 and the standard deviation was 0.1 for both. The length of the data series was 1500. The fit of the Gaussian mixture model to the histogram is shown in Fig. 1 along with the partitions and a small portion of the spectrum to demonstrate the symbolisation.

A typical example of a HMM inferred from the symbolised data is shown in Fig. 2. As can be seen, the generating and inferred model are very similar, with the correct architecture being inferred. To quantify this let us define the model distance, following Rabiner [27], as the difference in the log probabilities of the observed data, 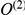, being generated by the generating model and the inferred model, designated 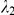 and 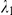 respectively, normalised for the length of the data, 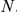


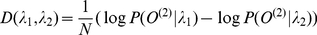
(3)


This measure is equal to zero for models with the same statistical properties. In our example the model distance is close to zero, 0.016, averaged over 5 repetitions, with a standard deviation of 0.009. The small error is due to the difficulty in estimating the exact distributions with data sets of this size. The methods are, therefore, capable of inferring accurate models under conditions typical to real data.

### Degenerate systems

**Figure 3:**
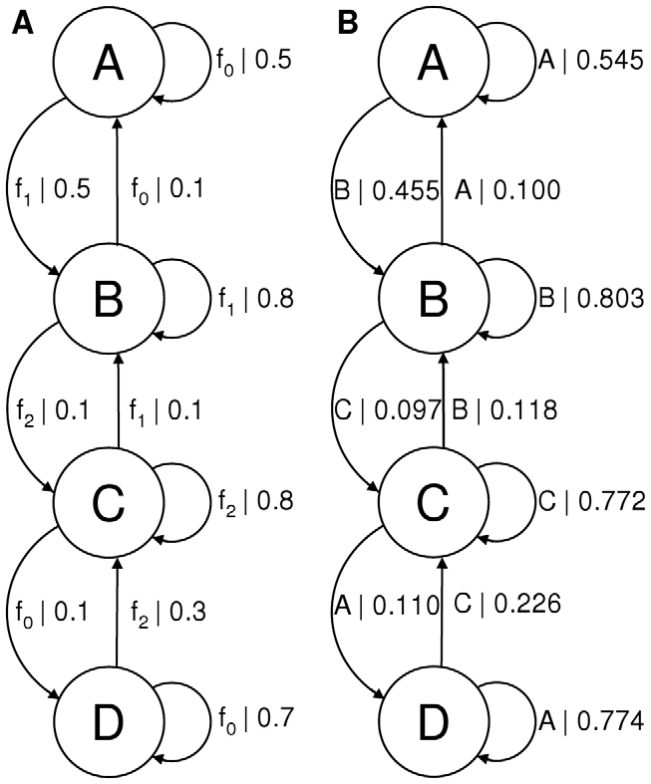
Comparison of generating and inferred HMMs with degenerate states. A) Model used to generate the data. This 4 state model has two states associated with the FRET level centred at 0.1 (denoted 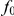) but with different probabilities of remaining in each state. B) The model inferred from the data. It has the correct architecture and the transition probabilities are close to those of the generating model. The model distance between the two is −0.42.

**Figure 4:**
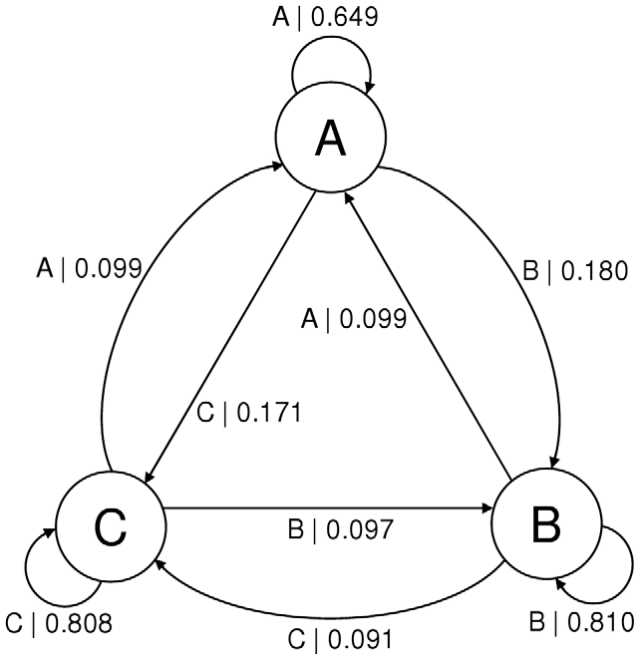
Model inferred with HaMMy. HaMMy cannot distinguish between the two degenerate states (A and D in Fig. 3A) resulting in a model with a state (labelled A) averaging the degenerate states' transition probabilities.

To demonstrate the ability of the methods to identify structure in data where different hidden states are associated with the same observable - degenerate systems - we also simulated data using the model shown in Fig. 3. Since this system is more complicated the data requirements to infer the correct architecture are comparatively higher; the result (also shown in Fig. 3) was obtained for 5000 data points. The Gaussian functions sampled on the transitions had means of 0.1, 0.5 and 0.9 and standard deviations of 0.09. In comparison, existing methods for inferring hidden Markov models from FRET data such as HaMMy, described in more detail below, may only hope to extract a 3 state model due to the constraint of associating each FRET level with one state. The ‘HaMMy’ programme was also run on this spectrum obtaining the 3 state model shown in Fig. 4. Note that one could identify states that had multiple transition rates associated with them by plotting histograms of the dwell times in each state as in the work by Laurens et al. [28]. The more recent method of Bronson et al. [29] is also capable of inferring degenerate models. We note however that, while it has fewer requirements of the data, it is more computationally intensive than the causal state methods, requiring 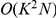 calculations as opposed to 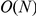 where 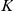 is the number of states and 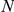 the number of observations.

### Experimental Data

Holliday Junctions are cross shaped, four way junctions of DNA and important intermediates in DNA recombination. As such they have been studied extensively [11], [30]–[32]. In the presence of divalent metal ions such as Mg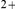 they have two stable conformations known as ‘stacked X’ conformers. Junctions will switch stochastically between the two conformations at a rate determined by the concentration of magnesium ions. If fluorescent probes are attached to the arms of the junction then these conformational changes may be observed by a change in FRET efficiency. Prior work has identified DNA sequences which form Holliday junctions with an approximately equal occupation of each conformer and characterised the dependency of the transition rate on the concentration of magnesium ions [33]. In order to test the methods on experimental data, these experiments were repeated and causal state models were successfully constructed from the resulting data.

### Experimental Methods

Biotin-labelled Holliday junctions (identical to ‘Junction 7’) were assembled and purified essentially according to published methods [33]. Equivalent junctions without donor and/or acceptor fluorophores were prepared in the same manner for use as controls. The junctions with only one fluorophore are used for collecting data with which to correct the FRET efficiency for overlap of the emission spectra of the two fluorophores. The junctions with no fluorophores are used to confirm a low level of background fluorescent contaminants. The junctions were bound to a cover glass (Menzel Glaser Nr 1.5) with a BSA-biotin streptavidin bridge using a modification of the method of McKinney et al. Briefly, the cover glass was cleaned with an argon plasma, then treated with biotinylated BSA (1 mg/ml, Sigma) for 5 minutes before washing extensively with T50 buffer (10 mM Tris-HCL [pH 7.5], 50 mM NaCl). Streptavidin (0.2 mg/ml, Invitrogen) was applied for 2 minutes before washing as before. A four channel imaging cell was constructed by sandwiching appropriately cut double-sided tape between the modified cover glass and a plasma-cleaned microscope slide. Holliday junctions (50 pM molecules) were added to the channel and incubated for 5 minutes before washing with T50 buffer supplemented with MgCl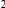 (as stated), an oxygen scavenger system (1 mg/ml glucose oxidase, 0.04 mg/ml catalase, and 0.8 mg/ml dextrose, Sigma) and anti-photobleaching reagents (1 mM methylviologen, 1 mM Ascorbic Acid, Sigma) [34].

**Figure 5:**
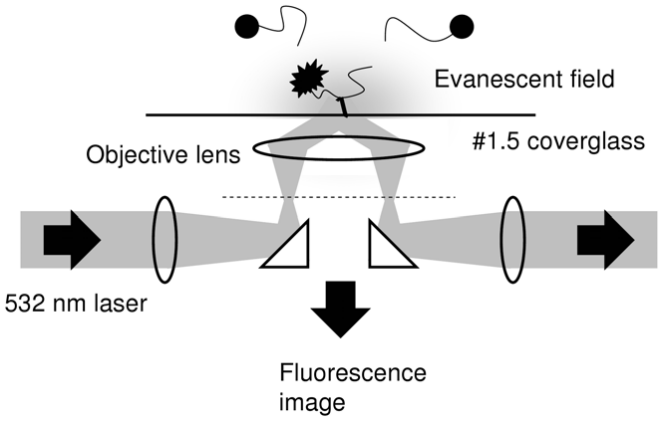
Schematic of the optical design for TIRF illumination.

FRET spectra were obtained using a custom built objective-based total-internal-reflection fluorescence (TIRF) microscope which is very similar in design to one described in detail elsewhere [35]. A schematic is shown in Fig. 5. Excitation was achieved using a 100 mW 532 nm laser (Laser Quantum, Ventus) attenuated by neutral-density filters. Emission light passed through a 532 nm notch filter (Semrock, StopLine) to remove scattered laser light and then a commercial dual-view system (Optosplit II, Cairn) to produce two images corresponding to the fluorescence from Cy3 (bandpass filter centred at 580 nm, width 60 nm) and Cy5 (bandpass filter centred at 655 nm, width 65 nm). Images were recorded using an electron-multiplied charge-coupled device (EM-CCD, iXon Du 897, Andor Technologies) with the Solis software package (Andor Technologies). For each dataset, the brightest objects were identified in each channel, matched between channels and the intensity time series extracted. Where these time series showed anticorrelation over a long period, FRET efficiencies were calculated according to methods in Ha [7] which includes a correction for leakage of the Cy3 emission into the Cy5 channel. Each FRET spectrum was then discretised using the methods described above and passed to the CSSR algorithm to construct causal state models. The models were then used with the transition probabilities to calculate the average transition rate for the junctions for each concentration. The spectra were also analysed using the ‘HaMMy’ programme as described [36]. Thirty nine spectra of varying lengths were obtained for a range of different magnesium ion concentrations.

#### HaMMy Results

Briefly, the HaMMY programme works in the following way, for more detail the reader is referred to the original paper [11] and references therein. First the user specifies the number of states (FRET levels) they wish to fit to the data. This determines the number of parameters in the model. These parameters are then varied in order to maximise the likelihood of observing the data using Brent's algorithm, a multi-dimensional optimisation algorithm. At each step in Brent's algorithm, i.e. for each set of parameter values, the likelihood of the data is calculated using the Viterbi algorithm (an efficient method, guaranteed to find the most probable state sequence). Providing the procedure does not converge to a local maximum rather than the global maximum it should infer the model with maximum likelihood of generating the data. Then one can examine the fitted spectrum and identify and eliminate extraneous states if they are never, or very infrequently, visited. Since we may identify and remove extraneous states but not add more, it is prudent when initially specifying the number of states to overestimate (by two as a rule of thumb).

Following this, the programme was run first of all with four states. Frequently this resulted in three FRET levels being visited in the idealised spectrum, two FRET levels very close together where one would assume there was only one, a case of the algorithm converging to a local maximum since the initial conditions were such that two FRET levels were equidistant from the actual FRET level and so both converged upon it. To circumvent this problem, initial guesses were supplied to the algorithm close to the actual FRET levels. The remaining spectra were fitted in this way. The HaMMy programme was able to infer a two state model for all of the spectra; extra states were hardly ever visited and for the most part had unphysical FRET values greater than one.

#### Causal State Modelling Results

The Causal State Modelling algorithms were also run on the data. It was found that although the requirements of the data for these methods were more stringent they could be successfully applied in the majority of cases.

The parameters of the inference algorithm were determined as follows. The significance level for the statistical test was set at 0.05. Then entropic considerations as to the likelihood of statistical fluctuations significant at this level guide an appropriate choice of maximum subsequence length. Since in these spectra data are relatively scarce, especially if the spacing of the FRET levels means a low percentage of the data are used, the maximum subsequence length was typically low, specifically 2. For longer spectra this was increased where possible.

Two-state models were inferred for thirty of the thirty nine spectra. Of those that failed seven were due to the FRET levels being too close together. In these cases, there were insufficient ‘certain’ data after the discretisation to be able to infer a model. Of these seven, in two borderline cases a model was inferred but the transition architecture was incorrect. In the remaining two cases the failure was due to the FRET levels changing monotonically with time so as to cross the partitions meaning no transitions between ‘certain’ symbols could be observed and hence no model inferred.

It was also found that, due to the high level of noise and the slight changes of FRET level with time leading to a higher weight between the two peaks, the routine often inferred a mixture model with more than two components despite the histogram of the FRET efficiencies clearly having two peaks. This may also have been due in part to the integration time of the camera averaging over transitions between states. In these cases, where two components were a more appropriate representation, the routine was constrained to fit the mixture model as such. Note that this constraint has no bearing on the number of states in the HMM which is still unconstrained.

**Figure 6:**
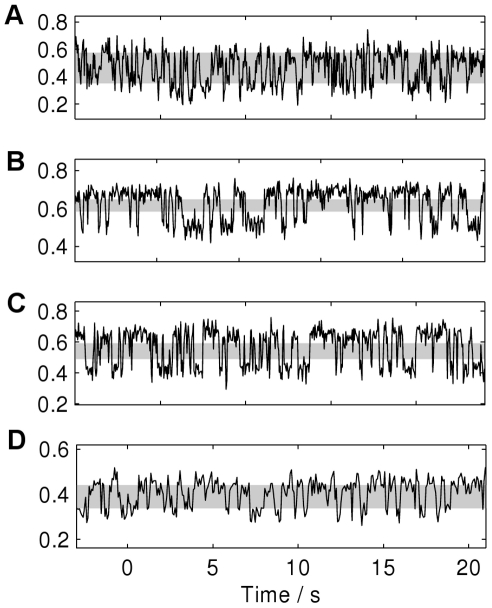
Example sections of FRET spectra. Mg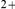 concentrations are A) 30 mM, B) 40 mM, C) 50 mM and D) 60 mM. The shaded region corresponds to the uncertain partition.

**Figure 7:**
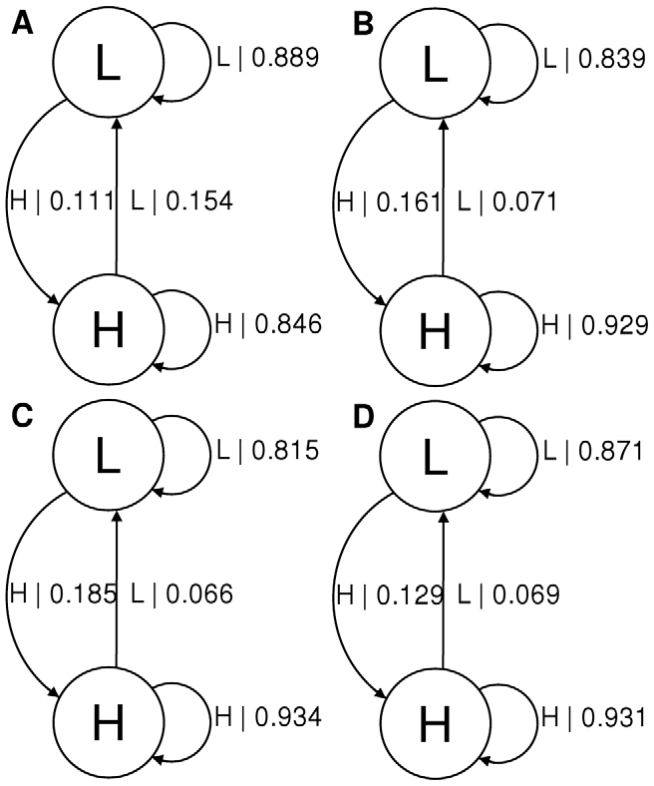
Causal state machines corresponding to the 4 spectra shown in **Fig. 6**. Mg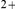 concentrations are A) 30 mM, B) 40 mM, C) 50 mM and D) 60 mM. Note that the actual transition rates are given by dividing transition probabilities by the sampling rate of the data, these were 41 ms per point for 30–50 mM and 71 ms per point for 60 mM.

Despite the problems outlined above, the methods performed well for the less noisy spectra of reasonable length. In Fig. 6 some example spectra are shown along with the resultant causal state models in Fig. 7.

## Discussion

### Method Comparison

The two methods are both capable of inferring models in agreement with our understanding of the physical system generating the data, but make different assumptions and have different requirements of the data and different model spaces (HaMMy's model space is contained in our method's model space). The speed of the two methods is comparable. Run time is typically less than 30 s on a desktop computer for both methods.

HaMMy requires as an input the number of FRET levels the user believes are present in the spectrum (overestimated to ensure the procedure is not constrained to fit a sub-optimal model) and assumes a model architecture with a state corresponding to each FRET level. Additional inputs specifying initial parameter values close to true values may improve the performance of the algorithm.

The causal state methods require (in the case of noisy data) the number of FRET levels the user believes are present, and a significance level at which to test whether or not distributions are equivalent. This significance level along with the quantity of data determines the remaining parameter, the maximum length of subsequence examined. The causal state methods make no assumptions regarding the model architecture but increase the number of states in the model if the current model cannot adequately account for structure in the data. They also allow for degeneracy, more than one state associated with the same FRET level. Both methods assume stationarity. As seen from the results above, the causal state methods have more stringent requirements regarding the quantity and quality of data. However, if a hidden state is suspected, this method is required.

**Figure 8:**
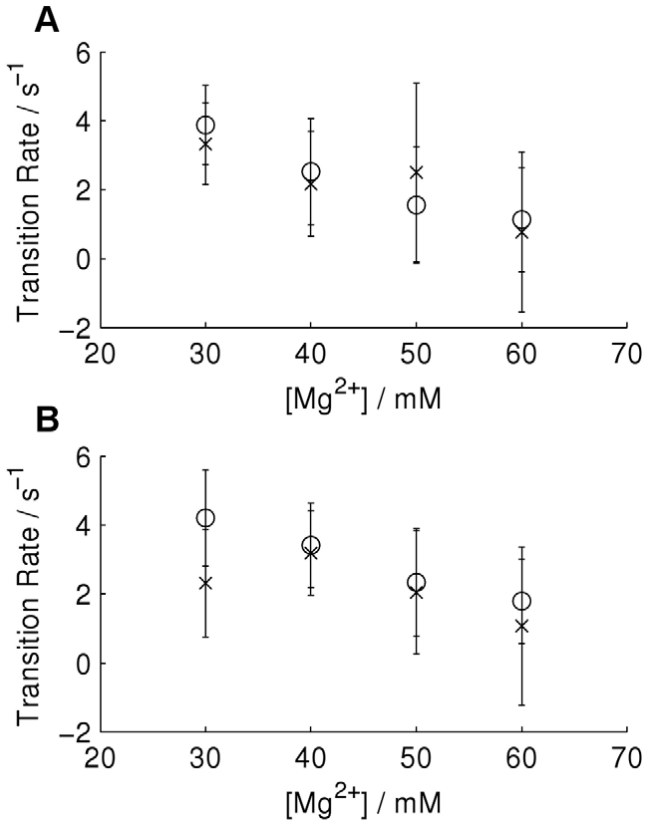
Average transition rates as a function of magnesium ion concentration. A) shows transition rate from the high FRET state to the low FRET state and B) the low FRET state to the high FRET state, with rates calculated using HaMMy (circles) and the causal state method (crosses). The error bars indicate the standard deviation.

The transition rates as a function of Mg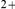 concentration are shown in Fig. 8 for both analysis methods. Note that these values are average results for multiple spectra, obtained by taking logs, calculating the mean and standard deviation for these transformed values, then exponentiating [11]. These values are in good agreement with previous work [33], exhibiting the same trend and being of the same order of magnitude; exact values for transition rates may vary with temperature. The values from the two different methods are consistent with each other in that the differences between them are within the error tolerances, however, we observe that the results from CSSR are consistently lower than those from HaMMY. We believe this is due to the causal state modelling underestimating the transition probabilities for the following reason. Since the data are time binned, all transitions must occur within an integration period resulting in a value of FRET efficiency for that bin which has been averaged to some extent. Due to the partitioning and discretisation scheme, these time averaged bins are more likely to be discounted by the causal state inference algorithm since they are more likely to fall in the ambiguous region between the two peaks in FRET efficiency. This introduces a bias into the statistics since time bins containing no transitions are less likely to be discounted in this way. For high data sampling rates relative to the time scale upon which the transitions occur this bias will be negligible, however, if the sampling rate is too low then the bias will become significant, as is the case for the rate inferred for the 30 mM magnesium ion concentration data. Since the simulated data was not subjected to further sampling or coarse graining this biasing was not observed and the correct transition probabilities were inferred.

### Conclusions

This paper presents a new method for inferring hidden Markov models from noisy time series, demonstrating the ability to infer the correct model architecture with minimal initial assumptions. We emphasise that the method is not only applicable to FRET spectra, but to any data source with a natural tendency to cluster such as that reported by other groups [37], [38]. It will generate unique, optimal and minimal predictors with only 2 input parameters. Application to the conformational dynamics of Holliday Junctions has demonstrated the ability of the methods to extract models from experimental data which agree with previous work in both model architecture and transition rates. The method provides a complementary alternative to existing methods of fitting HMMs to FRET spectra. Comparison between the new method and an existing maximum likelihood method shows that the requirements for the new method are more stringent; requiring a sufficient spacing of FRET levels, a sufficient quantity of data and a high sampling rate relative to the timescale of the dynamics of interest. However, since this new technique extends the model space and is able to directly discern multiple states with the same FRET distribution it holds a considerable advantage over its predecessor.

## Supporting Information

Figure S1 **A short section of the spectrum simulated using the model shown in** **Fig. 3** **of the main paper and the Gaussian functions there described.**
(TIF)

Figure S2 **A (normalised) histogram of the FRET efficiencies of the simulated spectrum with the fitted mixture model overlaid.**
(TIF)

Figure S3
**The partition boundary locations and the numbering of the partitions used to discretise the data.** The distributions are labelled 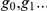 from left to right, the partitions are labelled 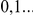 from left to right and the partition boundaries are labelled 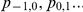 from left to right.(TIF)

Figure S4 **The shaded regions show the fraction of each model component which is associated with the certain region.** The smallest is found (in this case the central component) and then the partition boundary locations are adjusted in order to equalise them. The original partition boundary locations are indicated with solid black lines. The adjusted locations are indicated with dashed red lines.
(TIF)

Figure S5 **A short section of a FRET spectrum with calculated most probable trajectory.**
(TIF)

Figure S6 **Histogram showing the frequencies of dwell times for the low FRET state and a fitted exponential distribution.**
(TIF)

Figure S7 **Histogram showing the frequencies of dwell times for the high FRET state and a fitted exponential distribution.**
(TIF)

Table S1 **Word frequencies.**
(PDF)

Table S2
**The causal states and their assigned strings for** 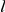 **= 1.**
(PDF)

Table S3
**The causal states and their assigned strings for** 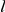 **= 2.**
(PDF)

Table S4
**The causal states and their assigned strings for** 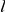 **= 3.**
(PDF)

Text S1 **Supporting Information providing an example demonstrating the discretisation methods, the proof of unbiased sampling, a walk through of the CSSR algorithm and a discussion of the stationarity assumption.**
(PDF)
