# Supplementary figures and images for: A New Method for Inferring Hidden Markov Models from Noisy Time Sequences
Source: PLoS One. 2012 Jan 11;7(1):e29703. doi: 10.1371/journal.pone.0029703 (PMC3256161; doi:10.1371/journal.pone.0029703)

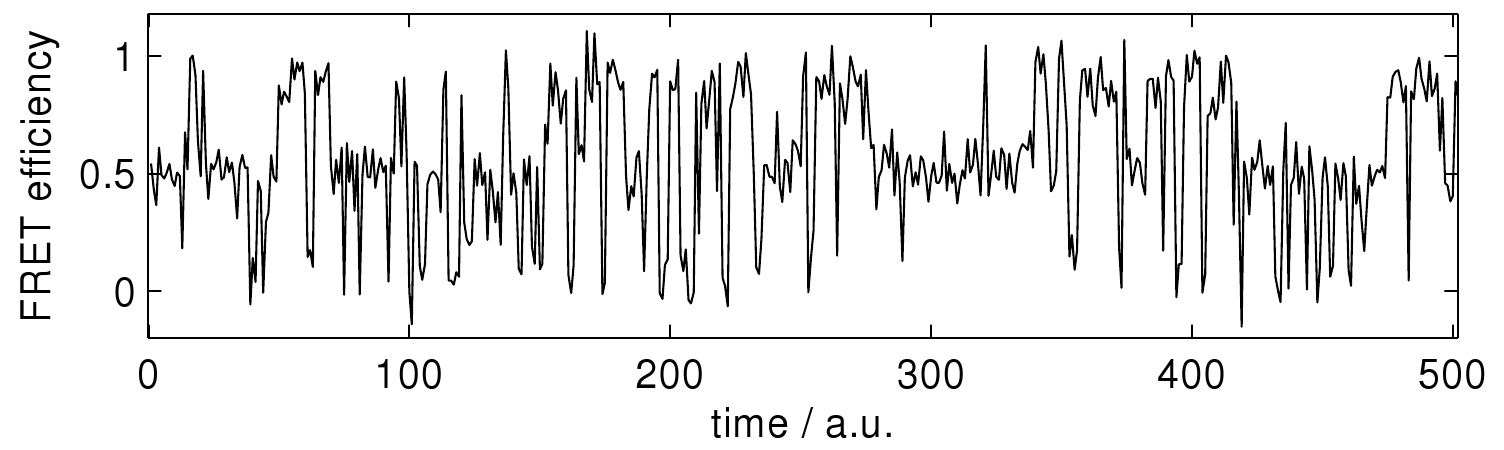

Supplement: Figure S1 — A short section of the spectrum simulated using the model shown in Fig. 3 of the main paper and the Gaussian functions there described. (TIF) [file pone.0029703.s001.tif]

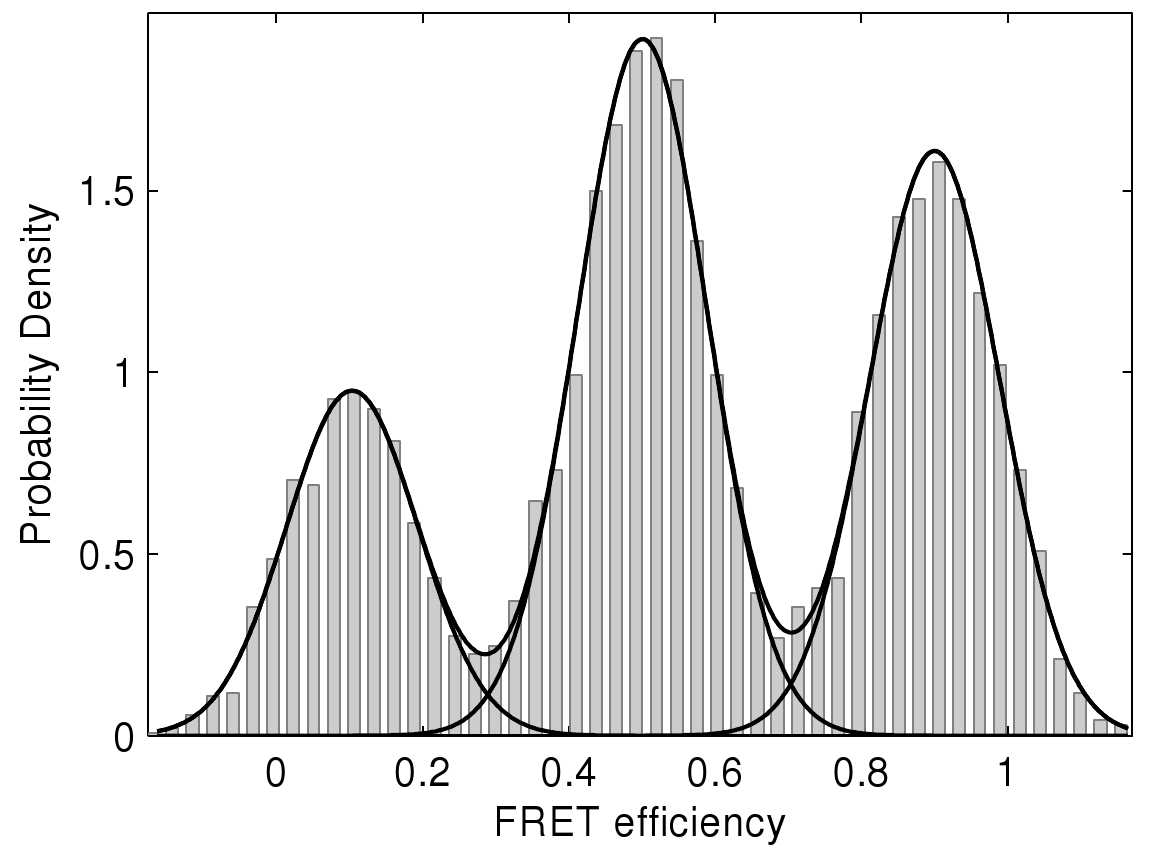

Supplement: Figure S2 — A (normalised) histogram of the FRET efficiencies of the simulated spectrum with the fitted mixture model overlaid. (TIF) [file pone.0029703.s002.tif]

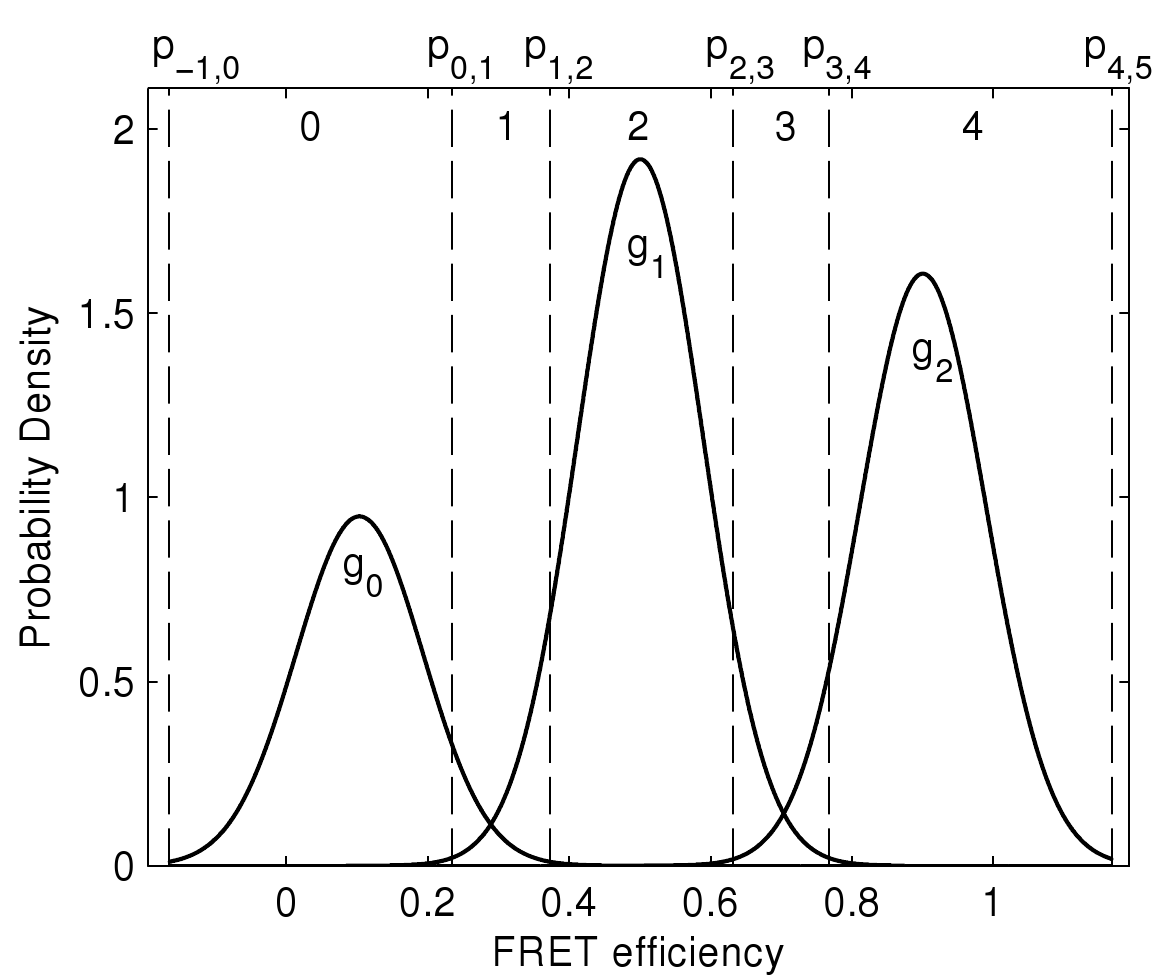

Supplement: Figure S3 — The partition boundary locations and the numbering of the partitions used to discretise the data. The distributions are labelled from left to right, the partitions are labelled from left to right and the partition boundaries are labelled from left to right. (TIF) [file pone.0029703.s003.tif]

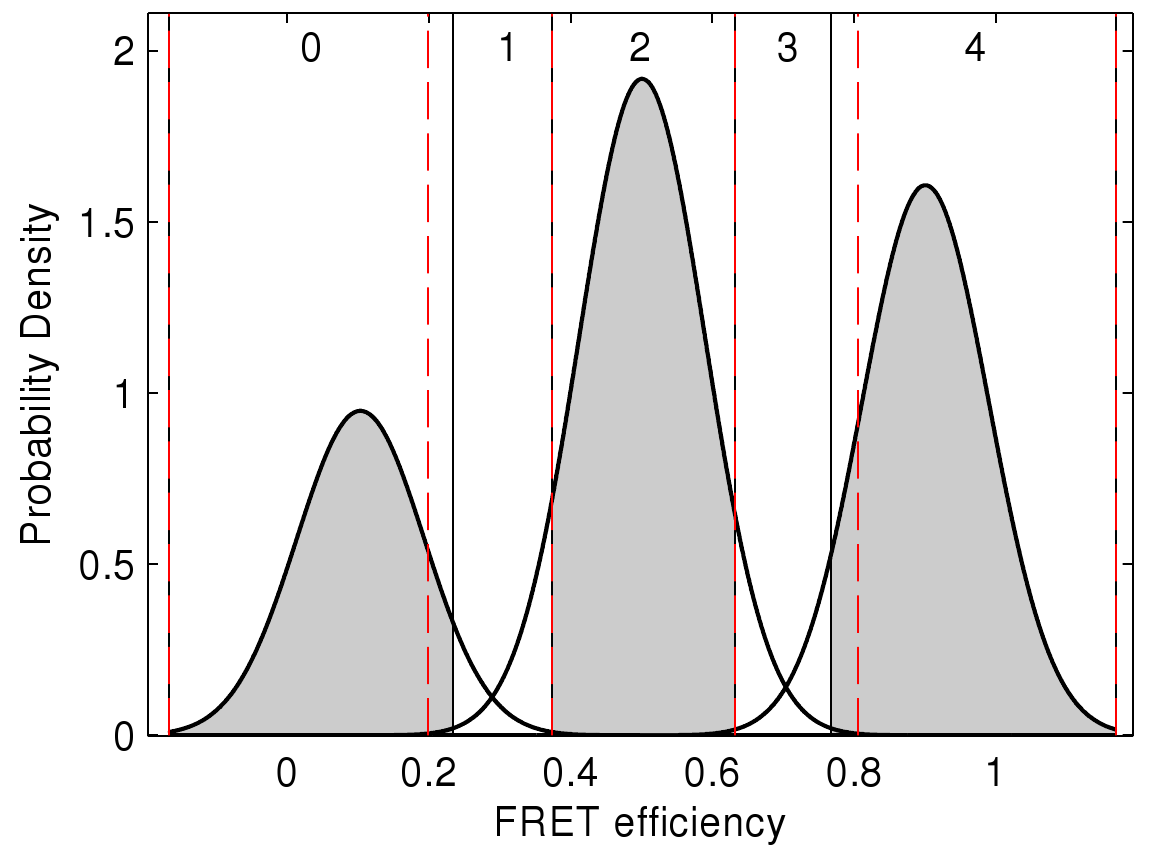

Supplement: Figure S4 — The shaded regions show the fraction of each model component which is associated with the certain region. The smallest is found (in this case the central component) and then the partition boundary locations are adjusted in order to equalise them. The original partition boundary locations are indicated with solid black lines. The adjusted locations are indicated with dashed red lines. (TIF) [file pone.0029703.s004.tif]

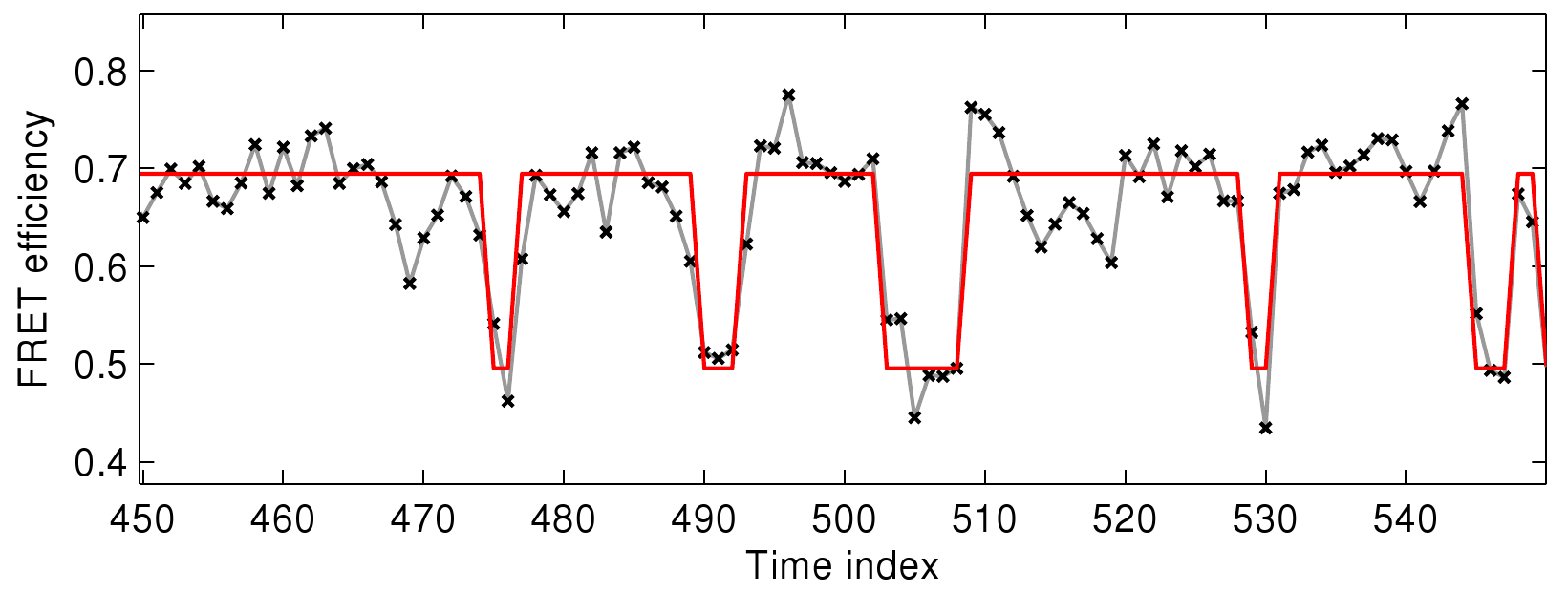

Supplement: Figure S5 — A short section of a FRET spectrum with calculated most probable trajectory. (TIF) [file pone.0029703.s005.tif]

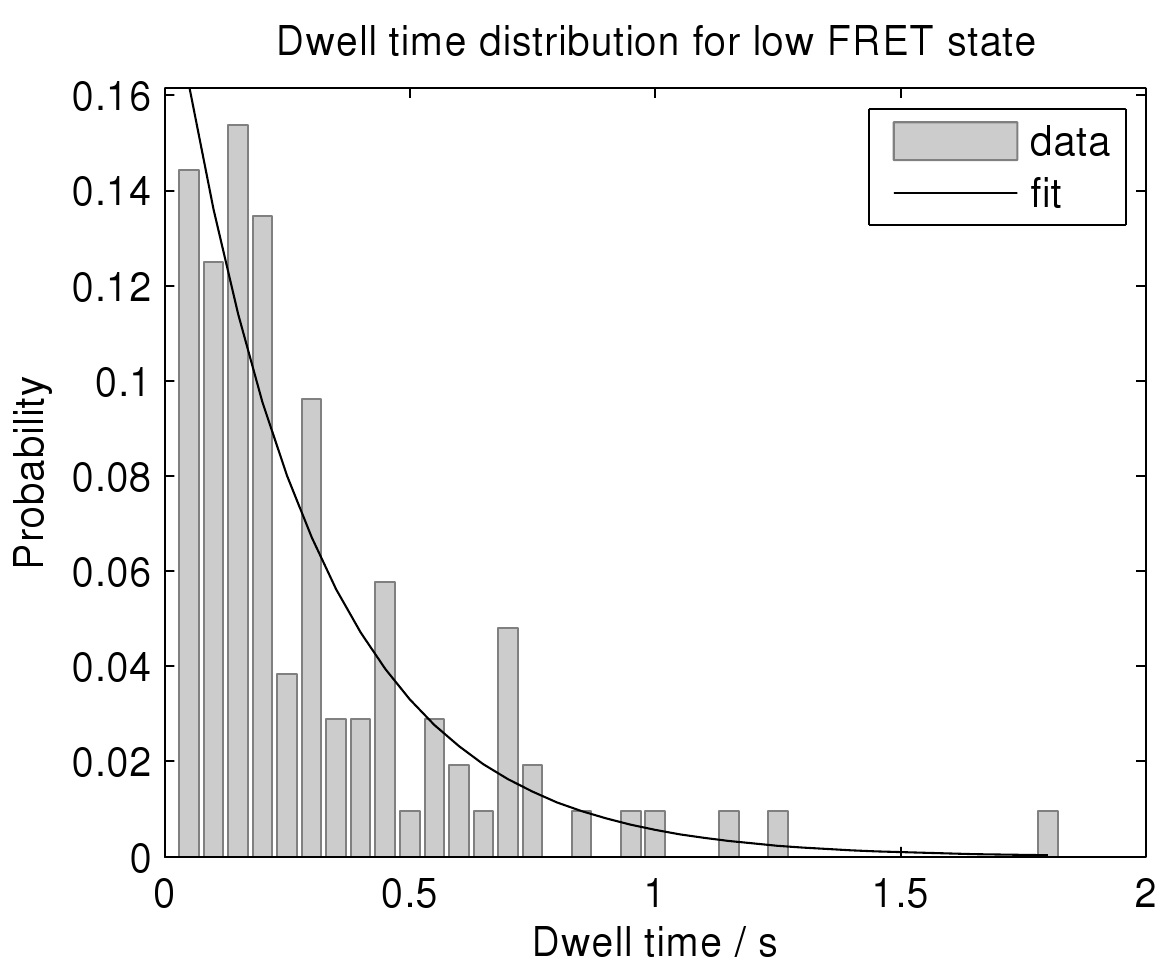

Supplement: Figure S6 — Histogram showing the frequencies of dwell times for the low FRET state and a fitted exponential distribution. (TIF) [file pone.0029703.s006.tif]

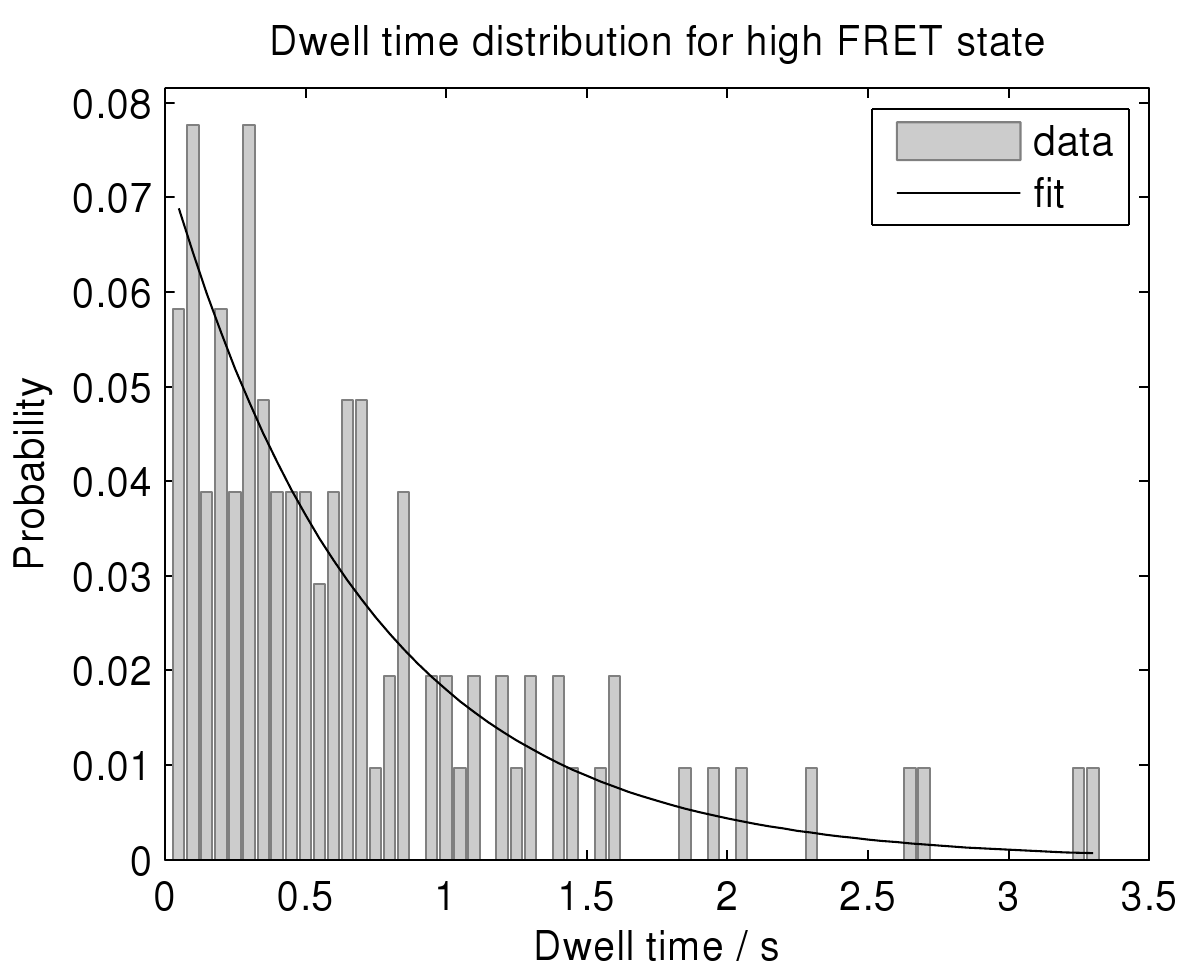

Supplement: Figure S7 — Histogram showing the frequencies of dwell times for the high FRET state and a fitted exponential distribution. (TIF) [file pone.0029703.s007.tif]
