# Supplementary material for: A New Method for Inferring Hidden Markov Models from Noisy Time Sequences
Source: PLoS One. 2012 Jan 11;7(1):e29703. doi: 10.1371/journal.pone.0029703 (PMC3256161; doi:10.1371/journal.pone.0029703)
Supplement: Table S1 — Word frequencies. (PDF) [file pone.0029703.s008.pdf]

Table 1: Word frequencies

Word	Frequency
0	925
2	1826
4	1540
00	516
02	150
04	136
20	151
22	1251
24	153
40	142
42	155
44	1014
