# Supplementary material for: A New Method for Inferring Hidden Markov Models from Noisy Time Sequences
Source: PLoS One. 2012 Jan 11;7(1):e29703. doi: 10.1371/journal.pone.0029703 (PMC3256161; doi:10.1371/journal.pone.0029703)
Supplement: Table S2 — The causal states and their assigned strings for = 1. (PDF) [file pone.0029703.s009.pdf]

Table 1: The causal states and their assigned strings for $l = 1$.

State 0	State 1	State 2
0	2	4
