# Supplementary material for: A New Method for Inferring Hidden Markov Models from Noisy Time Sequences
Source: PLoS One. 2012 Jan 11;7(1):e29703. doi: 10.1371/journal.pone.0029703 (PMC3256161; doi:10.1371/journal.pone.0029703)
Supplement: Table S4 — The causal states and their assigned strings for = 3. (PDF) [file pone.0029703.s011.pdf]

Table 1: The causal states and their assigned strings for $l = 3$.

State 0	State 1	State 2	State 3	State 4
0	2	4	20	40
00	02	04	200	400
000	22	24	020	040
	42	44	220	240
	002	004	420	440
	202	404		
	022	024		
	222	224		
	422	424		
	042	044		
	242	244		
	442	444		
