# Supplementary material for: A New Method for Inferring Hidden Markov Models from Noisy Time Sequences
Source: PLoS One. 2012 Jan 11;7(1):e29703. doi: 10.1371/journal.pone.0029703 (PMC3256161; doi:10.1371/journal.pone.0029703)
Supplement: Text S1 — Supporting Information providing an example demonstrating the discretisation methods, the proof of unbiased sampling, a walk through of the CSSR algorithm and a discussion of the stationarity assumption. (PDF) [file pone.0029703.s012.pdf]

A New Method for Inferring Hidden Markov Models from Noisy Time Sequences - Supporting Information

David Kelly[1,*], Mark Dillingham[2], Andrew Hudson[3], Karoline Wiesner[4]
1 School of Mathematics, University of Bristol, Bristol, UK
2 School of Biochemistry, University of Bristol, Bristol, UK
3 Department of Chemistry, University of Leicester, Leicester, UK
4 School of Mathematics, University of Bristol, Bristol, UK
∗ E-mail: dk3531@bristol.ac.uk

1 Example

The methods are illustrated using the example of the 4 state degenerate model used in the paper. A simulated 'spectrum' is generated using the model shown in Fig. 3 in the main paper. A short section of the spectrum is shown in Fig. S1.

Following the steps in the Methods section of the paper, a histogram of the spectrum FRET values (Fig. S2) is constructed. The Gaussian mixture models are fitted and the best selected using the Akaike information criterion (also shown in Fig. S2).

The space is partitioned by finding the permille quantiles (the points where there is a probability of 0.001 of seeing a value that or more extreme). The partitions are numbered from low to high FRET efficiency (starting at 0). As can be seen (Fig. S3), the even numbered partitions (0, 2 and 4) correspond to regions where there is (virtually) no overlap of the mixture model components. The odd numbered partitions (1 and 3) correspond to regions where there is overlap between mixture model components.

Now it is necessary to ensure that the fractions of probability mass associated with each of the certain regions for each mixture model component are the same. If they are not, more of one symbol will be discarded than of the others which will skew the distributions and bias the model. To do this the areas shaded in Fig. S4 and the fraction of the area of the entire component which this comprises are calculated.

Since the fraction for the middle mixture model component is the lowest the partitions are adjusted, as shown in Fig. S4 by the red dashed lines, to equalise the fractions for the other two model components. This ensures that using only the subset of the data which are certain does not bias the transition probabilities of the model since we discard an equal proportion of each symbol. This is proved mathematically below. The data are then discretised according to the partitions in which they fall and the discretised data passed to the CSSR algorithm.

2 Proof of unbiased sampling

The methods adjust the partitions such that the fraction of probability mass of each model component corresponding to the region of certainty is equal to the minimum fraction. Expressed mathematically,

$$\frac{A^{g_j}_{2j}}{A^{g_j}} = \min_i\left(\frac{A^{g_i}_{2i}}{A^{g_i}}\right)\forall j \tag{1}$$

where

$$A^{g_i}_{2i} = \int_{p_{2i-1,2i}}^{p_{2i,2i+1}} g_i(x)dx \tag{2}$$

and

$$A^{g_i} = \int_{-\infty}^{\infty} g_i(x)dx \tag{3}$$

Here the superscript refers to the distribution which is integrated in obtaining each area and the subscript (if present) indicates the partition boundaries which comprise the limits of the integral, with the n distributions numbered 0 to $n-1$ from left to right and the partition boundaries labelled with the numbers of the two partitions they separate as illustrated in Fig. S3. $A_{2i}^{g_i}$ is defined such that it corresponds to the area of the certain partition associated with the ith distribution. Note that this is equal to the joint probability that the symbol observed is $2i$ and the distribution which was sampled was g_i, $Pr(2i, g_i)$.

The probability of a data point sampled from a particular distribution, g_i, is given by

$$Pr(g_i) = \frac{A^{g_i}}{\sum_j A^{g_j}} \tag{4}$$

By substitution of $A^{g_j} = A_{2j}^{g_j} A^{g_i} / A_{2i}^{g_i}$ from Eq. 1 we obtain

$$Pr(g_i) = \frac{A_{2i}^{g_i}}{\sum_j A_{2j}^{g_j}} \tag{5}$$

which we recognise as the probability of observing the symbol associated with distribution g_i in the subset of the data from which the uncertain symbols have been eliminated. Therefore, the probabilities of the certain symbols are equal to the probability of the distributions with which they are associated, as required.

For longer symbol sequences the probability of their containing uncertain symbols is proportional to the length of the sequence. However, on average the same proportion of all of the different symbol sequences of a given length will contain uncertain symbols. Therefore, their relative frequencies will remain (approximately) equal to the probability of sampling the relevant sequences of distributions.

3 CSSR algorithm walk through

First the absolute frequencies of all the words containing only even number symbols are calculated. These are shown (up to word length 2) in Table S1.

The model is initialised with only one state containing only the null subsequence (that is conditioning on nothing, so the next symbol distribution associated with this state is just the frequency of symbols in the data). Then the length of subsequences included in the model, l, is increased from 0 to 1. Each symbol in the alphabet is then appended to the subsequences contained in the state, i.e. prefix '0', '2', or '4' to the null subsequence and examine the future distributions conditioned on these subsequences ('0', '2' and '4').

The first candidate subsequence is '0', so we look at the frequencies of the subsequences '00', '02' and '04'. These are then compared to the distribution of the existing state using the Kolmogorov-Smirnov statistical test. If the test is passed the subsequence '0' is added to the state containing the null subsequence. If the test is failed we create a new state for this subsequence.

This is done for all of the subsequences of this length. When there is more than one state then the distributions are compared with that of all of the states. If more than one state passes the test the subsequence is assigned to the state with the best match (highest p-value). Once all subsequences of this length are assigned to states the future distributions associated with the states are recalculated to include the future distributions conditioned on the newly added subsequences. The process continues until the maximum length of subsequence is reached.

The assignment of subsequences to the states for this example is shown in Tables S2 to S4 for each length of subsequence up to the maximum of 3.

The transitions between states are determined by appending symbols to the strings in that state. Since it is required that the machine is deterministic (being in a state and observing a particular symbol must uniquely determine the transition) it may be necessary to split the states. Therefore all of the states are examined, looking for conflicts where appending a symbol to different strings in the same state leads to more than one state. If a conflicting pair is found one is moved to a newly created state and the process is repeated. For this example the machine is deterministic as constructed and so no splitting of states is required.

The machine may contain transitory or synchronisation states. These are states which are only occupied when it is unknown which state of the machine is occupied. For this example State 0 is a transitory state (transitory states may not be returned to once left). It contains strings containing only '0's because when only these strings have been observed we are unsure whether the machine is in State 3 or State 4. Typically, only the recurrent part of the machine is required, therefore the transitory states are removed.

4 Stationarity Assumption

As already mentioned, the algorithm makes the assumption of stationarity. If one wishes to determine rate constants for the process under observation then this already implicitly assumes that the data will be treated as being stationary. There are two ways in which a process may not be stationary: static disorder and dynamic disorder.

Static disorder is observed in bulk systems where the rate constants for a process undergone by a population vary between different members. Single molecule experiments show this to be a common phenomenon, indeed it is one of the benefits of single molecule experiments that they can help to characterise the degree of static disorder.

Dynamic disorder is more problematic and is where the rate constants for a process vary as a function of time. Whether dynamic disorder is common is an open question.

These methods, like their predecessors, are insensitive to dynamic disorder. However, steps may be taken to check whether dynamic disorder is present and has invalidated the assumption of stationarity. Once the model for the data has been inferred in the manner described one may use the Viterbi algorithm to infer the most probable state sequence through the data and assign an idealised trajectory. This is the same method as employed by McKinney *et al* at each iteration of the likelihood maximisation procedure. An example section of the most probable trajectory calculated for one of the Holliday junction FRET spectra is shown in Fig. S5.

Once the state sequence has been determined histograms of the dwell times in each state may be plotted. If these histograms are well fitted by a single exponential decay then this indicates that the stationarity assumption is valid. In the case of non-stationary processes one would expect the histogram not to be fitted by a single exponential distribution but by a stretched exponential or multi-exponential distribution. In order to demonstrate this procedure the relevant plots for the Holliday junction spectrum are shown in Fig. S6 and S7. As can be seen, there is no compelling reason to believe the dwell times are not exponentially distributed, as may be expected for this simple system.
